# Validity and Repeatability of Single-Sensor Loadsol Insoles during Landing

**DOI:** 10.3390/s18124082

**Published:** 2018-11-22

**Authors:** Alexander T. Peebles, Lindsay A. Maguire, Kristen E. Renner, Robin M. Queen

**Affiliations:** 1 Kevin P. Granata Biomechanics Lab, Biomedical Engineering and Mechanics, Virginia Tech, Blacksburg, VA 24061, USA; hulbertk@vt.edu (K.E.R.); rmqueen@vt.edu (R.M.Q.); 2 Virginia Tech Carilion School of Medicine, Roanoke, VA 24018, USA; lmaguire@vt.edu

**Keywords:** plantar load, rehabilitation, return to sport, injury prevention, landing mechanics, symmetry

## Abstract

Clinically feasible methods for quantifying landing kinetics could help identify patients at risk for secondary anterior cruciate ligament injuries. The purpose of this study was to evaluate the validity and between-day repeatability of the loadsol insole during a single-hop and bilateral stop-jump. Thirty healthy recreational athletes completed seven single-hops and seven stop-jumps while simultaneous loadsol (100 Hz) and force plate (1920 Hz) measurements were recorded. Peak impact force, loading rate, and impulse were computed for the dominant limb, and limb symmetry was calculated between limbs for each measure. All outcomes were compared between the loadsol and force plate using intraclass correlation coefficients (ICC) and Bland–Altman plots. Fifteen participants completed a second day of testing to assess between-day repeatability of the loadsol. Finally, an additional 14 participants completed the first day of testing only to assess the validity of the newest generation loadsol, which sampled at 200 Hz. At 100 Hz, validity ICC results were moderate to excellent (0.686–0.982), and repeatability ICC results were moderate to excellent (0.616–0.928). The 200 Hz loadsol demonstrated improved validity ICC (0.765–0.987). Bland–Altman plots revealed that the loadsol underestimated load measures. However, this bias was not observed for symmetry outcomes. The loadsol device is a valid and repeatable tool for evaluating kinetics during landing.

## 1. Introduction

Screening for deficits in landing mechanics is important for both injury prevention as well as recovery [1,2,3,4,5,6,7]. Stiff landings, defined by high peak impact forces, are able to prospectively predict primary anterior cruciate ligament (ACL) injuries in adolescent athletes [1,2]. When returning to a sport following an ACL reconstruction and 6–12 months of physical therapy, athletes have significant side-to-side asymmetries, characterized by offloading the surgical limb and overloading the non-surgical limb [4,5,6,7]. High kinetic asymmetry in athletes returning to a sport following an ACL reconstruction is prospectively associated with an increased likelihood of sustaining a second ACL injury to the surgical or contralateral limb [3]. Embedded force plates are currently the gold standard for evaluating landing kinetics. However, due to cost and spatial requirements, this technology is not feasible for use in most clinical environments. One of the biggest challenges is therefore developing sensitive and clinically feasible methods to quantify deficits in landing mechanics in clinical environments.

Load sensing shoe insoles may be a good alternative to embedded force plates for measuring impact forces during landing. Pressure-sensing insoles have been previously used to assess running and cutting mechanics outside of a laboratory setting [8,9], and to improve walking mechanics in clinical populations [10]. Previous results indicate that pressure sensing insoles provide valid estimates of peak impact force, loading rate, and impulse relative to force plates during running [11,12]. However, as impact forces are much higher when landing from a jump than they are when running [9], it is currently unclear if in-shoe based sensors can provide valid estimates of kinetic outcome measures during landing. Additionally, while pressure-sensing insoles have been shown to be repeatable for measuring peak force and peak pressure during over-ground walking between days [13,14], the between-day repeatability of using load-sensing insoles during landing is unknown.

The loadsol (Novel Electronics, St. Paul Minnesota) is a single-sensor insole which is lightweight and cheaper than alternative collection methods, and should be simple to implement during an outpatient clinic visit. The loadsol consists of a single capacitive force sensor that covers the entire plantar surface of the foot. The main advantage of the loadsol over other in-shoe measurement systems is that it is wireless, and able to capture and transmit data to a mobile device via Bluetooth. Due to the dynamic nature of evaluating landing mechanics, it is important that participants are able to move without the impact of cables or large battery packs, which are required when using other load sensing insoles [8,9]. The purpose of this study is therefore to test the validity of the loadsol relative to gold-standard embedded force plates, and to assess the between-day repeatability of the loadsol. The single-leg single hop for distance and bilateral stop jump are widely used to evaluate landing mechanics [4,6,7]. Therefore, the validity and repeatability of the loadsol will be tested during these landing tasks.

## 2. Materials and Methods

Thirty recreational athletes (13 male/17 female; age 22.2 ± 3.4 years; height 1.72 ± 0.08 m; weight 66.4 ± 10.3 kg) were recruited, signed Virginia Tech institutional review board approved consent (VT IRB 16-614), and participated in the study. Inclusion criteria required all participants to be between the ages of 18 and 30, and to be recreationally active for at least 30 minutes three times per week. Participants were excluded if they had a history of major lower extremity surgery, had sustained a lower extremity injury in the previous two months, had a preexisting condition that limited participation in physical activities, or were pregnant. Of the 30 participants, 15 (8 male, 7 female) completed a second day of testing to assess between-day repeatability of the loadsol.

All participants were fitted with a pair of single sensor insoles (loadsol, Novel Electronics, St. Paul, MN, USA) and were provided a pair of neutral running shoes (Air Pegasus; Nike Inc., Beaverton, Oregon) to use during testing. Following manufacturer calibration procedures, participants were asked to walk around the laboratory for five minutes to become comfortable with wearing the insoles. To begin calibration, each participant was weighed using an embedded force plate (AMTI, Watertown, MA, USA), and the weight was entered in Newtons (N) into the loadsol application (app) on an iPad. The resolution was set to 5/10 N for a range of 0–2550 N, with the sampling rate set at the sensors’ maximum capacity of 100 Hz. The insoles were then calibrated through a series of three cycles of unloading the insoles and loading the plantar surface with the participant’s full body weight in single-leg stance. Calibration was tested by collecting a short trial of data for a single limb stance of each limb. The calibration was accepted and saved if it returned a bodyweight measurement of ±5% of the entered bodyweight. The calibration procedure was repeated if measurements were outside of the acceptable range. The dominant limb was defined as the preferred limb for kicking a soccer ball. In addition to using the insole sensors, ground reaction forces were collected using two tri-axial embedded force plates collecting at 1920 Hz (AMTI, Watertown, MA, USA) [4,6,15,16].

Each participant completed seven repetitions of a single-leg single hop for distance on each leg and seven stop jumps. For the single hop for distance, participants were required to hop forward for a maximal distance and land with their foot fully on a single force plate. In order to ensure the participants would land on the force plate and were hopping for maximum distance, each subject first performed two single hops for maximum distance. The average distance of these hops was used to determine the starting position for the single hops onto the force plate. A cone was placed at the starting position and participants were instructed to hop as far as possible without worrying about landing on the force plate. A trial was considered successful if the subject’s foot landed fully on the force plate, and if the landing was maintained for two seconds without the other foot touching down, the touching down of either hand, or a loss of balance. If unsuccessful, the trial was repeated. Participants completed single hops until seven successful trials were collected on each leg. After the completion of the single-leg hops, participants performed seven repetitions of a stop jump task. For the stop jump task, participants ran straight forward for five steps before taking off on one foot, of their choice, and landing on two feet, one foot on each force plate, and then immediately jumped back up as high as possible [15,16]. Participants were asked to run as quickly as possible into the jump and then to jump as high as possible. In order to minimize fatigue, a rest period of 30 seconds was provided between repetitions, and a two-minute rest period was provided between jumping tasks. Restrictions were not placed on arm movement during either jumping task, and no additional instructions were given on how to jump or land. Finally, in order to examine between-day repeatability of the loadsol, the above protocol was repeated for 15 participants, an average of eight days after their initial visit.

**Figure 1:**
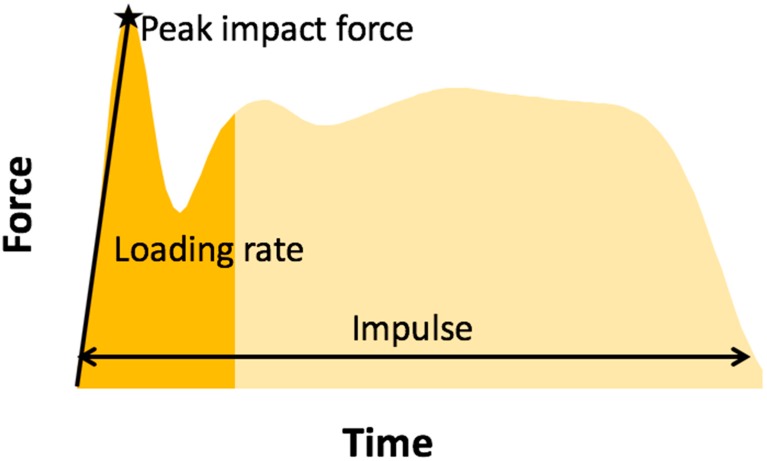
Example force–time data for one limb, collected during a stop jump trial.

Peak impact force, loading rate, and impulse were calculated from both the force plates and the loadsol sensors (Figure 1). Data analysis was performed using MATLAB (Version 9, The Mathworks, Inc, Natick, MA, USA). Ground reaction forces were filtered using a fourth-order Butterworth low-pass filter with a cutoff frequency of 15 Hz. For the stop jump trials, impulse was calculated as the area under the force-time curve from initial contact (first point when the force exceeded 10 N) to toe off (last point before the force dropped below 10 N) of the first jump [15]. Peak impact force was calculated as the largest peak in the first 25% of stance phase, and loading rate was calculated as the peak impact force divided by the time it took to reach the peak impact force [15]. For the single-hop trials, impulse was calculated for the first 200 milliseconds following initial contact. Pilot data indicated that peak knee flexion occurred at an average of 200 milliseconds after initial contact during a single hop (unpublished data); therefore, peak knee flexion was selected as the end of the landing phase. Peak impact force was taken as the maximum force during this time, and loading rate was calculated as the peak impact force divided by the time it took to reach the peak impact force following initial contact. All load outcome measures from both the force plate and the insole sensor were normalized to bodyweight for comparison. A limb symmetry index (LSI) was calculated as the ratio between the non-dominant and dominant limb for each trial, and then averaged together to obtain one symmetry value for each outcome measure (calculated with both the force plates and insole sensor) for both the single hop and stop jump tasks.

To test the validity of the loadsol for measuring landing kinetics, intraclass correlation coefficients (ICC) were calculated to compare the three outcome measures (impulse, peak impact force, and loading rate) for the dominant leg, as was the LSI of each outcome measure between the loadsol and force plate for both the stop jump and single-leg hop tasks. Next, the repeatability of the loadsol was tested using ICCs, comparing the outcome measures between the first and second collections. All ICCs were run as a two-way mixed model ICC (3,*k*) for consistency. ICC values greater than 0.9 were defined as excellent, between 0.9 to 0.75 as good, between 0.75 to 0.5 as moderate, and less than 0.5 as poor [17]. Finally, the bias of the loadsol to under- or overestimate force plate-derived load measures was visualized using Bland–Altman plots, and statistically tested using paired *t*-tests and 95% limits of agreement [18]. Significance was set at 0.05, and all statistical analysis was performed using SPSS (Version 24, SPSS Inc., Chicago, IL, USA).

During the course of this investigation, a new generation of the loadsol was released, which allowed data to be collected at 200 Hz. Due to the concerns that the 100 Hz insoles did not have a fast enough sampling rate for landing, we recruited a sample of 14 participants (8 male/6 female; age 23.1 ± 2.6 years; height 1.74 ± 0.04 m; weight 75.0 ± 13.9 kg) who met the same inclusion criteria, signed informed consent, and completed the first day of testing only. These data were collected to determine the impact of increasing sampling frequency on the validity of the loadsol sensor to capture impact forces. The same statistical analysis was performed to test the validity of the 200 Hz data relative to the force plates.

## 3. Results

Descriptive statistics, paired *t*-tests, ICC results, and confidence intervals for validity and repeatability of the loadsol sensors sampling at 100 Hz can be found in Table 1. At 100 Hz, the loadsol has moderate to excellent validity during the stop jump (ICC = 0.685–0.982) and poor to good validity during the single hop (ICC = 0.366–0.860) (Table 1). While the results indicate good to excellent between-day repeatability for the stop jump (ICC = 0.698–0.928) and single hop (ICC = 0.863–0.901) when looking at the dominant leg only, LSI outcome measures were poor to good for the stop jump (ICC = 0.004–0.297) and moderate to good for the single hop (ICC = 0.659–0.880). At 200 Hz, the loadsol insole had ­good to excellent validity during the stop jump (ICC = 0.821–0.987) and single hop (ICC = 0.756–0.949) for both dominant limb measures and symmetry measures (Table 2).

**Figure 2:**
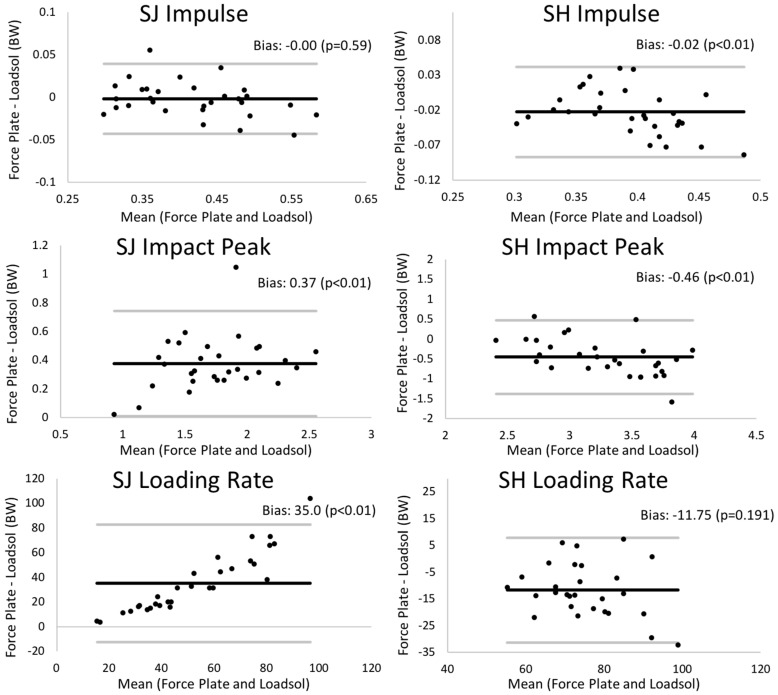
Bland–Altman plots showing the validity of the insole sensors at 100 Hz relative to the force plate. Note: on the plot, the bias (mean difference) and significance (paired *t*-test) of this bias are shown for each measure for both the stop jump (SJ) and single hop (SH) tasks. The bold black line on plots represents the mean bias, and the thin gray lines on plots are the limits of agreement (mean bias ±1.96 SD of bias).

**Figure 3:**
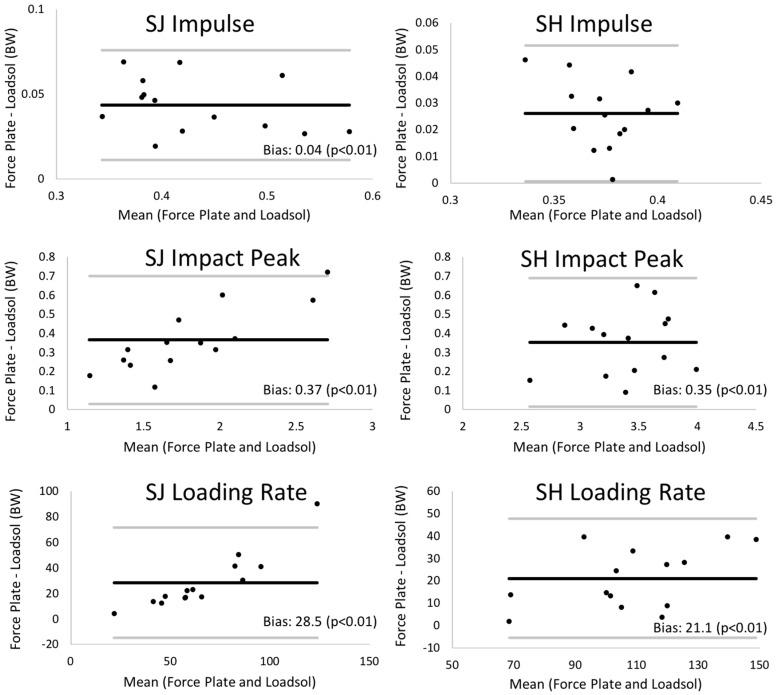
Bland–Altman plots showing the validity of the loadsol sensors at 200 Hz relative to the force plate. Note: on the plot, the bias (mean difference) and significance (paired *t*-test) of this bias are shown for each measure on both the stop jump (SJ) and single hop (SH) tasks. The bold black line on plots represents the mean bias, and the thin gray lines on plots are limits of agreement (mean bias ±1.96 SD of bias).

Bland–Altman plots comparing load outcome measures for the dominant limb between the loadsol at 200 Hz and the force plate can be found in Figure 2 and Figure 3. The insole and the force plate provided significantly different results when comparing dominant limb impulse and peak impact force, as well as loading rates during both the stop jump and single hop (all *p* < 0.01), where the insole provided smaller load measures than the force plate (Figure 1). When examining the symmetry measures, the insole and force plate were only different when comparing impulse LSI on the stop jump task (*p* = 0.03), where the insole overestimated the LSI. There were no differences between the insole and force plate for any other LSI outcome measure on the stop jump and single hop tasks (*p* > 0.16 for all).

## 4. Discussion

Clinically feasible methods to quantify landing mechanics deficits in athletes recovering from ACL reconstruction are currently lacking. The purpose of this study was therefore to examine the validity and repeatability of the loadsol single-sensor insole during a bilateral stop jump and a single-leg single hop for distance, both measures commonly used to assess recovery following ACL reconstruction. Generally, the results of the present study suggest that the loadsol is a valid and repeatable device to use in the assessment of ground-based kinetics and kinetic symmetry during hopping and jumping tasks. However, based on ICCs and Bland–Altman analysis, we suggest using insoles that are able to sample at 200 Hz or higher for measuring landing kinetics. Future work should explore the feasibility of using the loadsol in clinical environments and for identifying deficits in landing mechanics in clinical populations.

The validity and repeatability of ICC values reported in the present study are comparable to previous studies evaluating validity and repeatability of plantar pressure measuring insoles during walking and running [11,13,14,19]. Additionally, these results are comparable to other studies focused on clinical translation of force plate-derived outcomes [20,21,22,23]. For example, previous literature has shown that the Nintendo Wii (Redmond, WA, USA) balance board can be used as a surrogate for gold-standard force plates when measuring center of pressure oscillations [20,21]. Similarly, accelerometers have been shown to strongly agree with force plate-derived kinetic outcome measures during jumping and landing [22,23]. Though comparing the 100 Hz and 200 Hz loadsol sensors was not a direct purpose of the present study, our results do suggest that plantar loads should be collected at 200 Hz or higher for dynamic tasks, such as landing. Based on validity ICCs (Table 1 and Table 2), agreement between the force plate and loadsol was improved when collecting data at 200 Hz relative to 100 Hz for peak impact force, loading rate, and impulse on both the stop jump and single hop. Similar improvements were found when comparing load symmetry between the 100 Hz and 200 Hz insoles. Additionally, the 200 Hz insoles generally had smaller and more consistent 95% limits of agreement relative to the 100 Hz insoles. For example, the 95% limit of agreement for peak impact force on the single hop test at 100 Hz was −1.38 to 0.46, compared to 0.02 to 0.69 at 200 Hz, both represented in percent body weight. This finding demonstrates that the 200 Hz insoles consistently underestimated the load, while the 100 Hz insoles sometimes over- or underestimated the load. These findings are supported through previous literature, which has tested the effect of sampling frequency (25–500 Hz) on power and force measurements during a counter-movement jumping task [24]. Based on the repeatability ICCs and percent difference from reference values, the authors of that study conclude that a sampling rate of 200 Hz or higher is needed to evaluate kinetic outcome measures during the counter-movement jump [24]. In the present study, the force plates were sampled at 1920 Hz, which is seen as high enough to capture landing kinetics and has been used previously to evaluate landing kinetics [4,6,15,16].

Even when sampling at 200 Hz, there was a significant bias, with the loadsol insole underestimating force plate-based measures (Table 1 and Table 2). The underestimation bias of the loadsol for measuring force was expected, given the fact that the midsole of running shoes are viscoelastic and known to absorb energy during impact [25]. Previous studies have also observed this underestimation bias when comparing in-shoe pressure insoles and force plate-based ground reaction forces during running [11,12]. For most outcome measures, the bias appeared to be consistent across the measurement range (e.g. stop jump impulse). However, this was not the case for peak impact force and loading rate on the stop jump task, where the amount that the loadsol underestimated the force plate appeared to increase with increasing load. As ICCs for peak impact force and loading rate on the stop jump were acceptable (0.964 and 0.821 at 200 Hz), this demonstrates that the insole and force plate generally agree. We therefore believe that the loadsol insole could be used to differentiate individuals with stiff versus soft landings; however, this should be tested through future work. While there was an underestimation bias present when comparing the load between the loadsol insole and force plate on the dominant limb, LSIs of load measures were similar between the loadsol and force plate. This demonstrates that the underestimation bias was consistent between limbs, which supports the use of the loadsol insole for evaluating side-to-side kinetic asymmetries during landing tasks.

Between-day repeatability ICC results demonstrate moderate to excellent repeatability for assessing landing kinetics of the dominant limb (ICC > 0.698); however, symmetry repeatability ICCs ranged from poor to good (ICC = 0.004–0.880). These findings likely reflect between-day variation in task performance rather than sensor performance. To test this assumption, we ran between-day repeatability ICCs for load symmetry on the stop jump task using the force plate data, and found similar results (ICC = 0.137–0.580). Additionally, previous reports have found between-day ICC values of 0.69 for the peak impact force of a drop vertical jump task [26]. However, a between-day ICC of 0.962 was reported for a stop jump task that standardized jump height to 80% of participants’ maximum vertical jump height [27], which demonstrates that standardizing task performance may be crucial for test–retest repeatability. While higher between-day repeatability may have been achieved with a different landing task, the stop jump was chosen based on previous literature, which has used the stop jump to understand landing mechanics deficits in patients with ACL reconstruction [15,16]. To the authors’ knowledge, no previous studies have investigated the repeatability of kinetic measures on the single hop test or the repeatability of jump landing symmetry. The present study evaluated symmetry in healthy recreational athletes who had average LSIs close to perfect symmetry (100%), which likely influenced the low repeatability we observed. Future studies should determine if kinetic symmetry is more repeatable in pathological populations. 

There were several limitations in the present study. The motivation for the present study was to identify a clinically feasible method for quantifying landing kinetics, in order to identify landing mechanics deficits in patients with an ACL reconstruction (ACLR) prior to releasing them to return to their sport. However, the present study did not test the feasibility of using the loadsol in a clinical setting, and did not include patients with ACLR. Due to concerns about potential variability in landing patterns in ACL injury patients that could impact the outcomes, future work is needed to test whether or not the loadsol insole is feasible for clinical use, and whether or not these sensors are able to identify deficits in landing mechanics in ACLR patients. Another limitation is that when this investigation began, the loadsol insole was only able to sample at 100 Hz, which proved to be too low for accurate measurements of landing kinetics. However, an additional 14 participants were tested while wearing the newest generation of the loadsol insole, which sampled at 200 Hz. This secondary analysis revealed that the new 200 Hz insoles rendered superior validity ICCs, and should be used for measuring landing kinetics. While it is speculated that between-day repeatability would also improve at 200 Hz, this was not explored in the present study, and the repeatability of the 200 Hz insoles remains unknown.

## Figures and Tables

**Table 1:** Validity and repeatability of loadsol insole sensors during the stop jump and single hop tasks, collected at 100 Hz.

	**Stop Jump Validity**				**Single Hop Validity**			
	**FP**	**LS**	**ICC** **(95% CI)**	**Bias** **(95% LoA)**	**FP**	**LS**	**ICC** **(95% CI)**	**Bias** **(95% LoA)**
**IMP** **(BW)**	0.42 (0.08)	0.42 (0.08)	0.982 (0.96, 0.99)	0.00 (−0.04, 0.04)	0.38 (0.04)	0.40 (0.05)	0.860 (0.71, 0.93)	−0.02 * (−0.08, 0.04)
**IP** **(BW)**	1.93 (0.42)	1.55 (0.37)	0.941 (0.88, 0.97)	0.37 * (0.01, 0.74)	3.05 (0.37)	3.51 (0.60)	0.707 (0.38, 0.86)	−0.46 * (−1.38, 0.46)
**LR** **(BW)**	69.8 (33.2)	34.8 (11.3)	0.685 (0.34, 0.85)	35.0 * (−12.6, 82.6)	73.9 (20.6)	80.1 (14.2)	0.773 (0.51, 0.90)	−11.8 (−31.4, 7.93)
**IMP LSI** **(%)**	99.1 (13.2)	100.6 (16.0)	0.967 (0.93, 0.98)	−1.56 (−11.9, 8.8)	98.9 (5.0)	97.0 (9.2)	0.366 (−0.33, 0.70)	1.93 (−16.2, 20.0)
**IP LSI** **(%)**	97.5 (22.3)	98.3 (25.5)	0.965 (0.93, 0.98)	0.73 (−18.0, 16.5)	99.3 (9.5)	96.0 (15.3)	0.754 (0.48, 0.88)	3.31 (−18.9, 25.5)
**LR LSI** **(%)**	95.6 (26.7)	97.5 (23.8)	0.897 (0.78, 0.95)	−1.90 (−32.2, 28.4)	104.2 (20.0)	99.6 (21.1)	0.838 (0.66, 0.92)	2.22 (−23.9, 28.3)
	**Stop Jump Repeatability**				**Single Hop Repeatability**			
	**Day1**	**Day2**	**ICC** **(95% CI)**	**Bias** **(95% LoA)**	**Day1**	**Day2**	**ICC** **(95% CI)**	**Bias** **(95% LoA)**
**IMP** **(BW)**	0.43 (0.08)	0.43 (0.08)	0.928 (0.79, 0.98)	0.01 (−0.08, 0.09)	0.43 (0.05)	0.43 (0.07)	0.901 (0.71, 0.97)	0.00 (−0.07, 0.07)
**IP** **(BW)**	1.48 (0.36)	1.43 (0.27)	0.698 (0.10, 0.90)	−0.05 (−0.64, 0.55)	3.68 (0.62)	3.62 (0.85)	0.876 (0.63, 0.96)	−0.06 (−1.04, 0.91)
**LR** **(BW)**	32.4 (10.2)	32.2 (13.1)	0.721 (0.17, 0.91)	−0.14 (−21.7, 21.4)	81.5 (16.7)	79.3 (23.9)	0.863 (0.59, 0.95)	−2.18 (30.2, 25.8)
**IMP LSI** **(%)**	102.0 (17.2)	101.4 (10.0)	0.616 (−0.15, 0.87)	−0.55 (−29.5, 28.5)	98.2 (11.5)	101.7 (12.0)	0.880 (0.64, 0.96)	3.42 (−11.6, 18.5)
**IP LSI** **(%)**	104.5 (19.4)	109.1 (13.9)	0.297 (−1.09, 0.76)	4.59 (−37.8, 47.0)	97.1 (14.3)	105.0 (15.6)	0.704 (0.12, 0.90)	7.98 (−20.0, 36.0)
**LR LSI** **(%)**	104.0 (24.0)	108.7 (17.5)	0.004 (−1.99, 0.66)	4.72 (−53.5, 63.0)	97.9 (19.0)	118.4 (37.3)	0.659 (0.02, 0.89)	20.55 (−38.0, 79.0)

Note: values presented as mean (SD) for force plate (FP) and loadsol (LS) on the top, and for Day1 and Day2 on the bottom. Intraclass correlation coefficient (ICC) 95% confidence intervals (CI) are shown below the ICC values. Fixed bias is shown as FP-LS for validity and Day2-Day1 for repeatability, with Bland–Altman 95% limits of agreement (LoA) shown below. IMP: impulse; IP: peak impact force; LR: loading rate; BW: body weight; LSI: limb symmetry index. * indicates significant (*p* < 0.05) difference between force plate and loadsol.

**Table 2:** Validity of loadsol insole sensors during the stop jump and single hop tasks collected at 200 Hz.

	Stop Jump Validity				Single Hop Validity			
	FP	LS	ICC (95% CI)	Bias (95% LoA)	FP	LS	ICC (95% CI)	Bias (95% LoA)
**IMP** **(BW)**	0.45 (0.07)	0.41 (0.07)	0.987 (0.96, 0.99)	0.04 * (0.01, 0.08)	0.39 (0.02)	0.36 (0.02)	0.872 (0.60, 0.96)	0.03 * (0.00, 0.05)
**IP** **(BW)**	1.98 (0.53)	1.62 (0.38)	0.964 (0.89, 0.99)	0.37 * (0.03, 0.70)	3.57 (0.41)	3.22 (0.37)	0.949 (0.84, 0.98)	0.35 * (0.02, 0.69)
**LR** **(BW)**	80.7 (36.4)	52.2 (16.4)	0.821 (0.44, 0.94)	28.5 * (−14.6, 71.5)	119.2 (26.9)	98.1 (20.2)	0.912 (0.73, 0.97)	21.1 * (−5.4, 47.7)
**IMP LSI** **(%)**	94.4 (13.7)	97.4 (14.9)	0.970 (0.91, 0.99)	−3.07 * (−12.6, 6.5)	101.9 (4.7)	103.7 (7.0)	0.765 (0.27, 0.93)	−1.77 (−11.9, 8.4)
**IP LSI** **(%)**	101.1 (23.0)	102.4 (21.7)	0.967 (0.90, 0.99)	−1.28 (−17.1, 14.5)	104.9 (13.8)	102.1 (11.2)	0.917 (0.74, 0.97)	2.79 (−10.8, 16.4)
**LR LSI** **(%)**	108.1 (33.2)	107.0 (30.4)	0.966 (0.90, 0.99)	1.09 (−21.4, 23.6)	110.7 (23.3)	104.1 (18.6)	0.756 (0.20, 0.93)	6.61 (−30.0, 43.2)

Note: values presented as mean (SD) for force plate (FP) and loadsol (LS) and ICC 95% confidence intervals are shown in parentheses below ICC values. Fixed bias is shown as FP-LS, with Bland–Altman 95% limits of agreement (LoA) shown below. IMP: impulse; IP: peak impact force; LR: loading rate; BW: body weight; LSI: limb symmetry index. * indicates significant (*p* < 0.05) difference between force plate and loadsol.
